# Assessing the Potential of Using the Langdon 5D(5B) Substitution Line for the Introgression of *Aegilops tauschii* Into Durum Wheat

**DOI:** 10.3389/fpls.2022.927728

**Published:** 2022-07-07

**Authors:** Manel Othmeni, Surbhi Grewal, Jack Walker, Cai-yun Yang, Ian P. King, Julie King

**Affiliations:** School of Biosciences, The University of Nottingham, Sutton Bonington Campus, Loughborough, United Kingdom

**Keywords:** *Aegilops tauschii*, introgression, durum wheat, hexaploid wheat, chromosome-specific KASP markers

## Abstract

*Aegilops tauschii*, the D-genome donor of hexaploid wheat, provides a source of genetic variation that could be used for tetraploid (durum) wheat improvement. In addition to the genes for wheat quality on the D-genome, which differentiate between bread and durum wheats in terms of end-use properties, genes coding for resistances to biotic and abiotic stresses are also present on the D-genome which would be useful in durum wheat. The introgression of *Ae. tauschii* into durum wheat, however, requires cytogenetic manipulation to induce homoeologous chromosome pairing to promote recombination. For this purpose, the introgression of *Ae. tauschii* into durum wheat was performed through a bridge cross of the wild species to the Langdon 5D(5B) disomic substitution line that lacks the *Ph1* locus present on chromosome 5B, followed by a cross of the F_1_ to the durum wheat cultivar Om Rabi 5. Subsequent generations were self-fertilized, and these were screened for D-genome introgressions using (i) D-genome-specific Kompetitive Allele-Specific PCR (KASP) markers and (ii) KASP markers polymorphic between the 5D chromosomes of wheat, present in the Langdon 5D(5B) substitution line, and of *Ae. tauschii*. Homozygous introgression lines were confirmed using genomic and fluorescence *in situ* hybridization. The results showed that the use of the Langdon 5D(5B) disomic substitution line did not promote D-genome introgression across all linkage groups with only a limited success in the introgression of *Ae. tauschii* 5D segments into durum wheat.

## Introduction

*Aegilops tauschii* is a diploid self-pollinating goatgrass species (2*n* = 14, DD) considered to be the D-genome donor of hexaploid wheat (*Triticum aestivum*; 2*n* = 6× = 48; AABBDD) (Dvorak et al., 1998). This species is widely distributed from northern Syria and Turkey in the west to western China in central Eurasia (van Slageren, 1994) with the center of origin thought to be in the western part of its range from where it expanded east (Matsuoka et al., 2008) showing adaption to very diverse environments such as desert margins, steppe regions, sandy shores, and even humid temperate forests (van Slageren, 1994). Several studies have shown the allelic diversity of *Ae. tauschii* to be greater than that present in the D sub-genome of hexaploid wheat (Reif et al., 2005; Cox et al., 2017). The studies have included a wide variety of molecular tools such as chloroplast DNA variation (Matsuoka et al., 2008, 2009; Takumi et al., 2009), AFLP (Mizuno et al., 2010), SSR (Naghavi and Mardi, 2010), isozymes (Dudnikov and Kawahara, 2006), and RAPD markers (Okuno et al., 1998), with the greater allelic diversity recently confirmed when the sequencing of 242 *Ae. tauschii* accessions showed that only 25% of the genetic diversity present in the wild species had contributed to the initial gene flow into hexaploid wheat (Gaurav et al., 2022).

The allelic diversity present within *Ae. tauschii* has been shown to encompass a wide range of resistances/tolerances to both biotic and abiotic stresses. For example, Jia et al. (2013) looked at the genome sequence of *Ae tauschii* and found 1,219 genes potentially involved in disease resistance and 485 genes potentially involved in abiotic stress tolerance including cold tolerance (216 genes) and drought tolerance (14 genes), while a more recent study using *k*-mer-based association mapping (Gaurav et al., 2022) also identified genomic regions harboring candidate genes for disease and pest resistance. This allelic diversity has been extensively used in bread wheat with the introgression of several genes of interest from *Ae. tauschii* either through the recreation of synthetic hexaploid wheat (SHW) that involves crossing tetraploid wheats with *Ae tauschii* followed by chromosome doubling or through the direct crossing of *Ae. tauschii* to bread wheat followed by backcrossing to bread wheat (reviewed in Cox et al., 2017). These traits include genes of resistance to several diseases (Ma et al., 1993; Eastwood et al., 1994; Miranda et al., 2006; Leonova et al., 2007; Mandeep et al., 2010; Olson et al., 2013), bread-making quality (Li et al., 2007), pre-harvest sprouting tolerance (Gatford et al., 2002; Imtiaz et al., 2008), and yield-related traits (Gororo et al., 2002; Rasheed et al., 2014).

The introgression of *Ae. tauschii* into durum wheat, however, is more complex compared with its introgression into bread wheat, as cytogenetic manipulation is required to enhance recombination and induce homoeologous chromosome pairing between the D-genome of *Ae. tauschii* and the A- and the B-genome chromosomes of durum wheat. Two strategies have previously been successfully used to introgress segments from wild relatives into durum wheat, the first being the use of the LND 5D(5B) disomic substitution line (Joppa and Williams, 1988) and the second the use of *ph1c* mutant durum lines (Giorgi, 1978, 1983). Due to a good compensation of chromosome 5D for 5B, the use of the 5D(5B) disomic substitution line has proved an efficient system to promote pairing in a number of interspecific hybrids involving durum wheat and different wild relatives, such as *Thinopyrum* spp., carrying genes for resistance to wheat rusts, barley yellow dwarf virus, and Fusarium head blight (Jauhar and Almouslem, 1998). In a comparison using genomic *in situ* hybridization (GISH), intergenomic pairing appeared to be higher when using the substitution line than the *ph1c* genotypes (Jauhar et al., 1999). The observed difference in the amount of pairing promotion might, in part, be attributable to the effect of chromosome 5D as this chromosome has been shown to carry a pairing promoter on its long arm, which in *T. aestivum* has been estimated to have a greater effect than the promoter on the 5BS arm (Sears, 1976).

To date, however, only a set of durum wheat/*Ae. tauschii* monosomic addition lines have been developed by Dhaliwal et al. (1990). The set initially comprised four whole D chromosomes (1D, 2D, 3D, and 6D) and three intergenomic translocation chromosomes (4DS−5DS, 5DL−7DS, and 7DL−4DL) which were then used to generate all 14 chromosome arms of the D-genome as monosomic additions. Despite the high fertility of these monosomic chromosome arm addition lines, data of Makino (1981) showing the low gametic transmission of the *Ae. tauschii* chromosomes indicated some difficulty in the feasibility of their maintenance and the isolation of the corresponding disomic additions.

The direct introgression of *Ae. tauschii* into durum wheat has not been used to any great extent. In fact, only a few genomic regions from the D-genome of bread wheat harboring genes of interest have been directly targeted and introgressed into durum. For example, the introgression of a 4DL chromosome segment from bead wheat carrying the *Kna1* locus conferred increased salt tolerance (Dvorák et al., 1994), and another introgression from chromosome 4D of bread wheat conferred aluminum tolerance in an elite durum wheat cultivar (Han et al., 2014). The introgressions from the D-genome of bread wheat provide a further indication of the potential of *Ae. tauschii* considering the greater allelic diversity it harbors.

The aim of this study was to introgress segments of the *Ae. tauschii* genome into the durum wheat cultivar “Om Rabi 5” using the Langdon disomic LND 5D(5B) substitution line to generate a panel of durum wheats with different D-genome segments introgressed, together with substitution and addition lines, to cover as much of the D-genome linkage groups as possible. In order to track and characterize the introgressions produced, D-genome-specific Kompetitive Allele-Specific PCR (KASP) markers were used and extra 5D-chromosome-specific KASP markers polymorphic between chromosome 5D of wheat present in the LND 5D (5B) substitution line and the 5D chromosome of *Ae. tauschii* were generated.

## Materials and Methods

### Plant Material and Crossing Scheme

**Figure 1:**
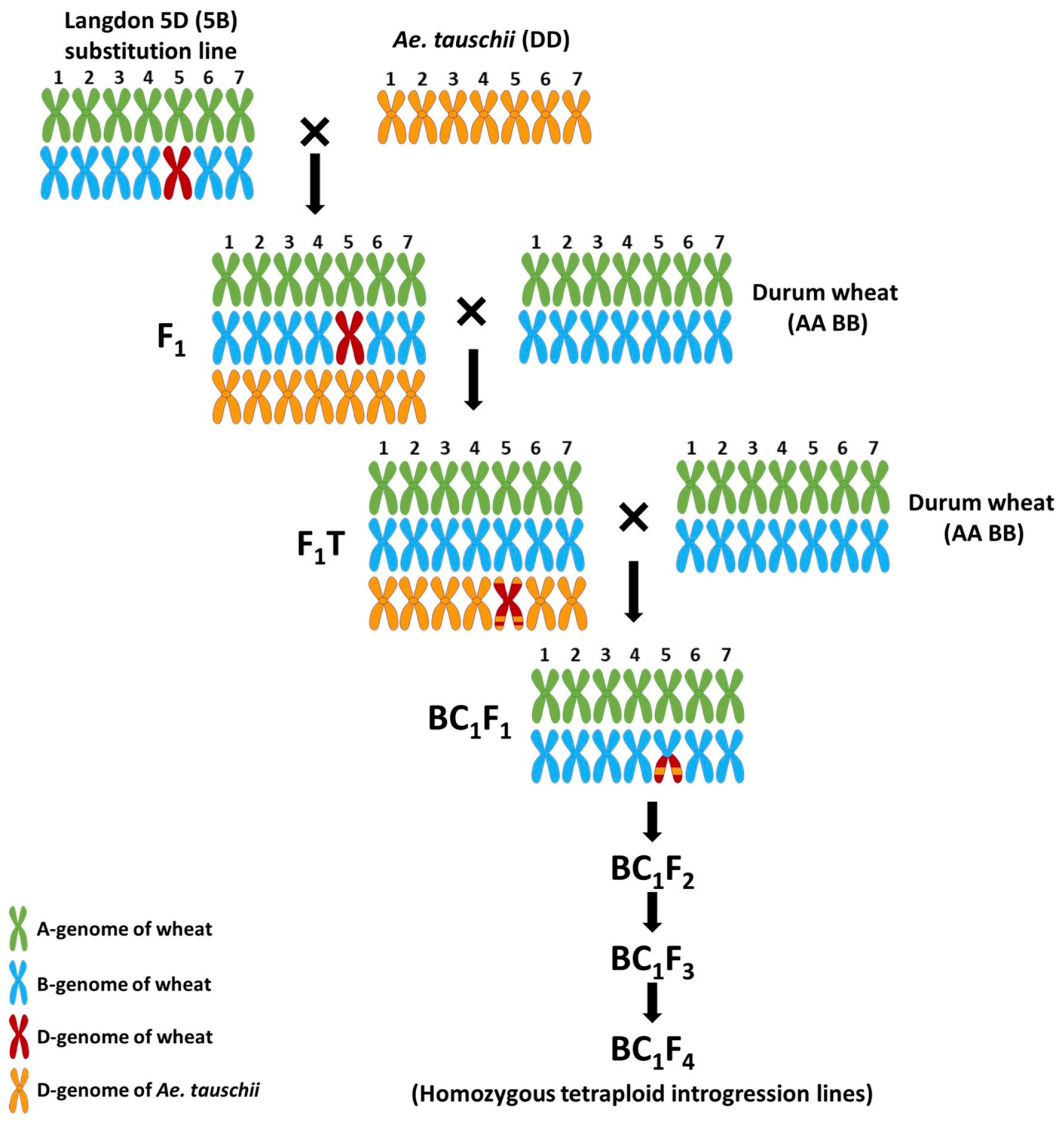
Crossing diagram of *Ae. tauschii* introgression into durum wheat.

The crossing program was carried out using *Ae. tauschii*, accession P99-95.1-1 [obtained from the United States Department of Agriculture (USDA)], the Langdon 5D(5B) disomic substitution line [LND 5D(5B)] (obtained from the USDA Fargo, North Dakota), and the durum wheat variety “Om Rabi 5” (obtained from ICARDA). *Ae. tauschii* was used as the pollen donor to cross with the LND 5D(5B) line (Figure 1). Twenty spikes of LND 5D(5B) were emasculated after the removal of the top and basal spikelets and the central florets of each remaining spikelet. Thus, only the two lateral florets of each spikelet were kept and individually pollinated with *Ae. tauschii* pollen, with an average of 28 florets pollinated per emasculated spike. The F_1_ seed produced was crossed and backcrossed as the female parent to the durum variety Om Rabi 5 generating the F_1_ top (F_1_T) and the BC_1_F_1_, respectively.

### Genotyping

Leaf material was collected from 10-day-old seedling plants, and DNA extraction was performed as described by Thomson and Henry (1995). BC_1_F_2_ plants and subsequent generations were genotyped using D-genome-specific Kompetitive Allele-Specific PCR (KASP™) markers, while BC_1_F_3_ plants and subsequent generations were also genotyped using 5B-chromosome-specific KASP markers (Grewal et al., 2020a). Lines showing the presence of D-genome chromosomes or segments were selected for further work. KASP markers, polymorphic between linkage group 5D of wheat and linkage group 5 of *Ae. tauschii*, were designed to identify *Ae. tauschii* 5D introgressions when recombined with the 5D of wheat initially present in the LND 5D(5B) substitution line. These markers were tested on the BC_1_F_3_ and subsequent generations. All primer details are given in Supplementary Material 1. The number of D-genome KASP markers used varied at each generation as more markers were designed for greater resolution (Grewal et al., 2020a).

Genotyping was carried out as described by Grewal et al. (2020b). PCRs contained 1 ng of genomic DNA, 2.5 μL of KASP reaction mix (ROX), 0.068 μL of primer mix, and 2.43 μL of nuclease-free water in a final volume of 5 μL set up using the automated PIPETMAX^®^ 268 (Gilson, UK). PCR conditions were set to 15 min at 94°C; 10 touchdown cycles of 10 s at 94°C and 1 min at 65–57°C (dropping 0.8°C per cycle); and 35 cycles of 10 s at 94°C and 1 min at 57°C,” and PCRs were performed in a ProFlex PCR system (Applied Biosystems by Life Technology). Fluorescence detection of the reactions was performed using a QuantStudio 5 (Applied Biosystems), and the data were analyzed using the QuantStudioTM Design and Analysis Software V1.5.0 (Applied Biosystems).

### Cytogenetic Analysis

#### Genomic *in situ* Hybridization

Slides of metaphase chromosome spreads were prepared as described in Kato et al. (2004) and King et al. (2017). Multi-color genomic *in situ* hybridization (Mc-GISH) was conducted using the labeled total genomic DNA of the three potential wheat progenitor species: the A-genome donor *T. urartu*, the B-genome donor *Ae. speltoides*, and the D-genome donor *Ae. tauschii*. DNA was extracted from young leaves using the CTAB method (Zhang et al., 2013) and labeled using the nick translation procedure (Luchniak et al., 2002): *T. urartu* was labeled with ChromaTideTM Alexa FluorTM 488-5-dUTP (Invitrogen; C11397; green), *Ae. tauschii* with ChromaTideTM Alexa FluorTM 594-5-dUTP (Invitrogen; C11400; red), and *Ae. speltoides* with DEAC-dUTP (Jena Bioscience; NU-803-DEAC; blue). Slides were hybridized with a probe mix containing 1.5 μL *Ae. urartu*, 3 μL *Ae. speltoides*, and 3 μL *Ae. tauschii* (a ratio of 1:2:2). Slides were counterstained with 4'-6-diamidino-2-phenylindole (DAPI) before being analyzed with the fully automated Zeiss Axio Imager.Z2 upright epifluorescence microscope (Carl Zeiss Ltd., Oberkochen, Germany). Images were taken using a MetaSystems Coolcube 1 m CCD camera and processed with ISIS (image processing) software (Metasystems GmbH, Altlussheim, Germany).

#### Karyotype Analysis Using Fluorescence *in situ* Hybridization

Slides of metaphases spreads were prepared as described above for multi-color fluorescence *in situ* hybridization (mc-FISH) analysis. Two repetitive genomic DNA sequences, namely, pSc119.2 (McIntyre et al., 1990) and pAs1 (Rayburn and Gill, 1986), were labeled by nick translation with ChromaTideTM Alexa FluorTM 488-5-dUTP (green) and Alexa Fluor 594-5-dUTP (red), respectively, and used as probes hybridized to the slides with a ratio of 1:1. Slide counterstaining with DAPI, image capture, and analysis were performed as described above for mc-GISH. Karyotype analysis was carried out by comparison to the karyotype for Chinese Spring (Tang et al., 2014).

## Results

### Population Generation

**Figure 2:**
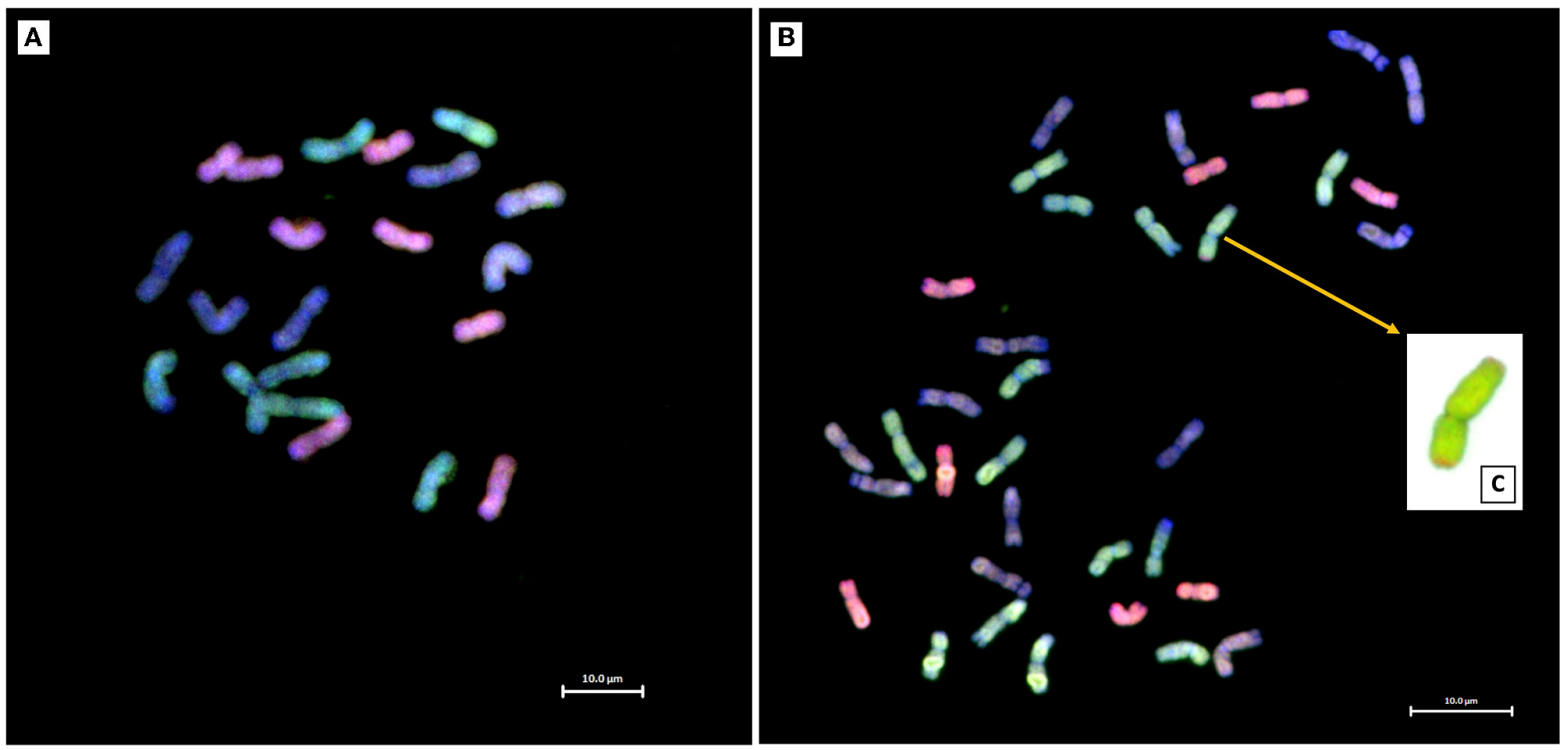
Mc-GISH of root-tip metaphase spreads of **(A)** the F_1_ and **(B)** F_1_T plants revealing the presence of a telomeric A-d chromosome translocation **(C)** in the F_1_T plant (A-genome, green; B-genome, blue/purple; D-genome, red).

Twenty crosses between *Ae. tauschii* and the LND 5D(5B) substitution line generated four amphihaploid F_1_ seeds, only one of which germinated and grew to maturity. The F_1_ amphihaploid was verified using mc-GISH which showed a total of 21 chromosomes: seven A-genome chromosomes, six B-genome chromosomes, and eight D-genome chromosomes including a pair of 5D chromosomes (Figure 2A). The F_1_ plant was male-sterile and was therefore crossed to Om Rabi 5 as the female parent generating only one F_1_T seed which germinated and grew to maturity. Mc-GISH of the F_1_T plant showed the presence of one genomic translocation that consisted of a small D-genome segment translocated in the telomeric region of an A-genome chromosome short arm designated as an A-d translocation (Figure 2C) as well as the retention of eight D-genome chromosomes (including two 5D chromosomes), together with 14 A-genome and 13 B-genome chromosomes in a total of 35 chromosomes (Figure 2B) with only one copy of chromosome 5B. The F_1_T plant was then backcrossed to the durum parent Om Rabi 5, and 13 BC_1_F_1_ seeds were obtained.

### Screening for *Ae. tauschii* Introgressions

**Figure 3:**
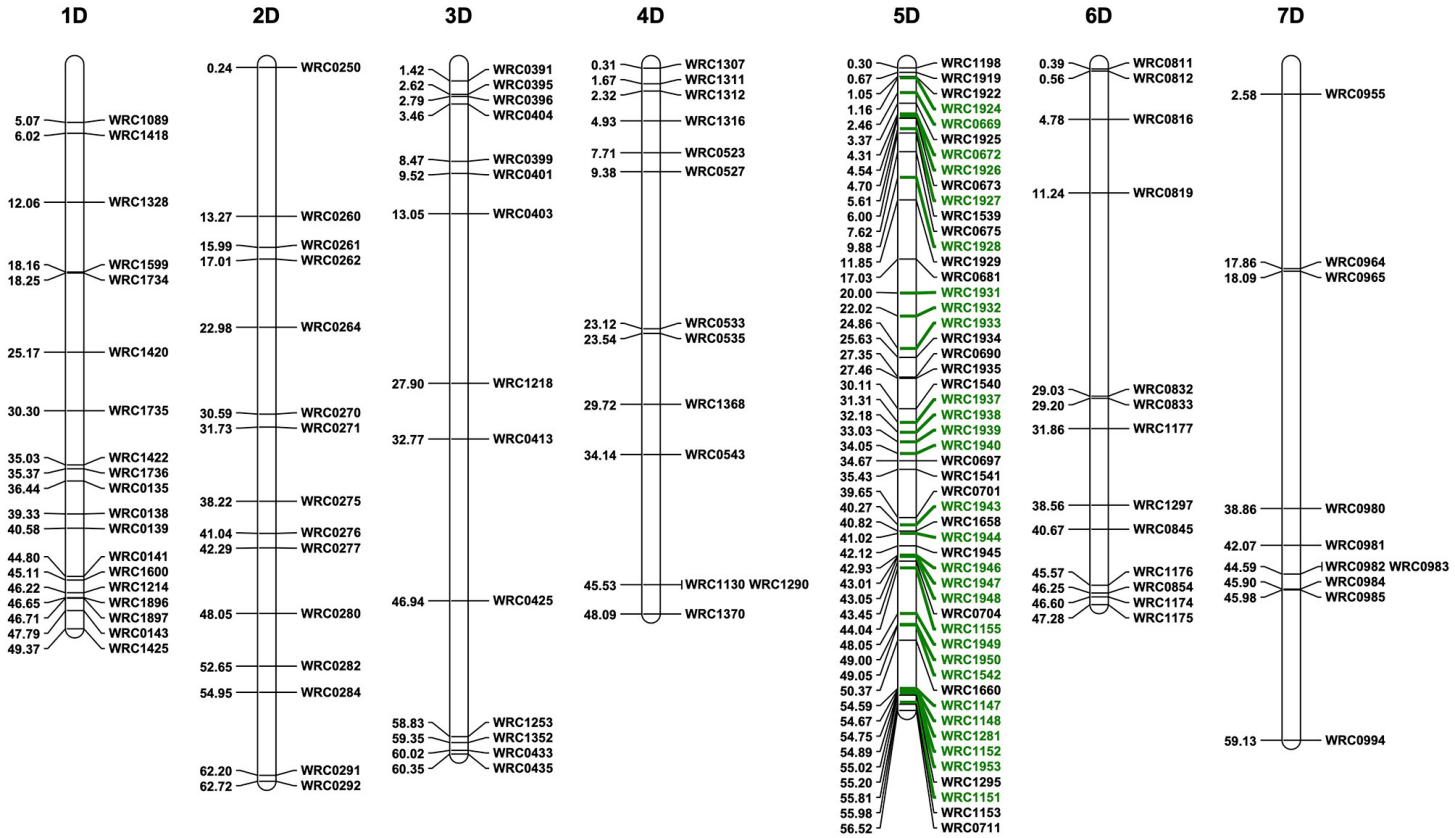
Physical position of the D-genome-specific KASP markers on the seven D-genome linkage groups in bp 10^−7^. Polymorphic KASP markers between Chinese Spring and *Ae. tauschii* for LG 5 are highlighted in green.

Eight of the 13 BC_1_F_1_ seeds germinated and grew to maturity. The BC_1_F_1_ plants were screened for the presence of the D-genome using the 136 D-genome-specific KASP markers (Figure 3). Plants containing D-genome introgressions in a total of 28 chromosomes were self-pollinated to obtain tetraploid homozygous introgression lines. To identify and select BC_1_F_1_ plants carrying a 5D chromosome which included 5D introgressions from *Ae. tauschii*, the BC_1_F_1_ plants were also screened with 28 KASP markers polymorphic between chromosomes 5D of Chinese Spring and *Ae. tauschii* (shown in green in Figure 3) as recombination between these chromosomes was expected during chromosome pairing in the F_1_ gamete.

KASP genotyping of the eight BC_1_F_1_ lines showed the decreased retention of D-genome chromosomes after backcrossing to the durum wheat parent. The number of D-genome chromosomes retained varied between one to three chromosomes with all lines retaining at least one copy of the 5D chromosome (Table 1). Mc-GISH confirmed the KASP results for the D-genome retention and again enabled the genomic composition of each line to be established together with the presence of genomic translocations. The total number of chromosomes varied between 28 and 31 with only 13 B chromosomes in all lines (due to monosomic 5B: Table 1). Four lines, namely, BC_1_F_1_-244, BC_1_F_1_-247, BC_1_F_1_-251, and BC_1_F_1_-252, retained only 5D, shown by mc-GISH analysis to be monosomic 5D(5B) substitution lines in a total of 28 chromosomes, while BC_1_F_1_-245 also retained only 5D as a monosomic 5D(5B) substitution but also carried the “A-d” translocation previously identified in the F_1_T line in a total of 29 chromosomes. Two further lines, namely, BC_1_F_1_-246 and BC_1_F_1_-252, showed the same “A-d” translocation, indicating its retention in 38% of the BC_1_F_1_ lines screened. The KASP genotyping of the three lines carrying the “A-d” translocation detected only 5D plus a 2D in line BC_1_F_1_-246, thus suggesting that the small telomeric D-genome segment is either a very small segment from linkage group 5 or a segment not covered by the KASP markers. Three BC_1_F_1_s carried other whole monosomic D-genome chromosomes (1D, 2D, and 4D—see Table 1) with one BC_1_F_1_ also carrying the long arm of chromosome 3D and shown with mc-GISH to be involved in a centromeric translocation with a B-chromosome long arm (3DL.BL) (GISH pictures in Supplementary Material 2).

**Table 1:** Genomic composition of the BC_1_F_1_ lines.

**Lines**	**A-chrom. No**.	**B-chrom. No**.	**D-chrom. No**.	Translocation*No. copy	**Total chrom. No**.	**D-genome KASP results**
BC_1_F_1_-244	14	13	1	0	28	5D
BC_1_F_1_-245	14	13	1	A-d*1	29	5D
BC_1_F_1_-246	14	13	2	A-d*1	29	2D, 5D
BC_1_F_1_-247	14	13	1	0	28	5D
BC_1_F_1_-248	15	13	2	0	30	4D, 5D
BC_1_F_1_-250	14	13	3	3DL.BL*1	31	1D, 4D, 5D
BC_1_F_1_-251	14	13	1	0	28	5D
BC_1_F_1_-252	13	13	1	A-d*1	28	5D

### Screening for D-Genome Chromosomes and Introgressions in Later Generations

Ten seeds were germinated from each of the eight BC_1_F_1_s with a total of 59 BC_1_F_2_ seeds germinating and reaching maturity. The number of KASP markers used to genotype this generation was reduced to 113 markers, with the elimination of markers for the D-genome linkage groups (6D and 7D) not detected in the BC_1_F_1_ generation. KASP genotyping showed that all BC_1_F_2_ lines from BC_1_F_1_-246 had lost all D chromosomes/segments, and therefore, these lines were not taken forward.

### Screening for 1D, 2D, 4D, and 3DL.BL Chromosomes

**Figure 4:**
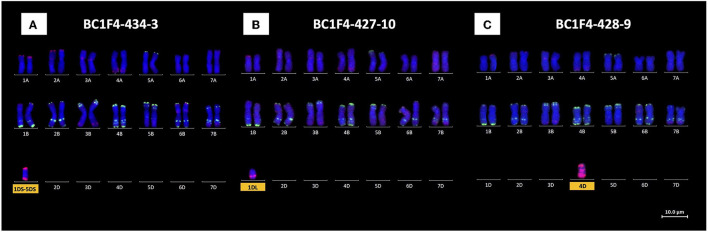
Mc-FISH-based karyotype using the Oligo-pAs.1 (red) and Oligo-pSc119.2 (green) probes counterstained with DAPI (blue) of the **(A)** 1DS.5DS translocated chromosome, **(B)** 1DL chromosome arm, and **(C)** 4D-chromosome monosomic addition lines.

Monosomic chromosome 1D, present in BC_1_F_1_-250, was found in two of the BC_1_F_2_ progeny together with monosomic 4D. KASP genotyping of a third line (BC_1_F_2_-250-3) with D-chromosome markers showed the presence of chromosome arms 3DL, 1DS, and 5DS. Mc-FISH analysis showed that 1DS and 5DS formed a 1DS.5DS translocated chromosome. All three lines were progressed to the BC_1_F_3_ generation where KASP genotyping showed two lines to be monosomic for 1DS.5DS (e.g., BC_1_F_3_-434-9) and a third line to contain chromosome arm 1DL (BC_1_F_3_-427-10). Further progression to the BC_1_F_4_ generation again failed to generate lines homozygous for either 1DS.5DS or 1DL. According to the mc-FISH-based karyotyping analysis of these lines, both segments were retained as monosomic and monotelosomic additions, respectively, with 28 AABB chromosomes (Figures 4A,B). Chromosome 2D, originally present in BC_1_F_1_-246, was lost as early as the BC_1_F_2_ progeny in all screened lines.

Chromosome 4D was shown to be present in two BC_1_F_1_ lines, namely, 248 and 250. Only one BC_1_F_2_ line from BC_1_F_1_-248 was shown to retain chromosome 4D as a monosomic addition together with 5D(5B) also as a monosomic substitution. KASP genotyping of six BC_1_F_3_ progeny from this BC_1_F_2_ line showed the presence of chromosome 4D in only one line, which was subsequently lost from the BC_1_F_4_ progeny. Three BC_1_F_2_ progeny from BC_1_F_1_-250 retained chromosome 4D: in the first plant as monosomic 4D and in plants 2 and 3 as a double monosomic (4D with 1D). These three BC_1_F_2_ lines were therefore taken forward to BC_1_F_3_ and, where KASP markers showed the presence of 4D, to BC_1_F_4_. Mc-FISH-based karyotyping analysis showed that chromosome 4D was retained as a monosomic addition in two BC_1_F_4_ plants (BC_1_F_4_-428-9, Figure 4C), and therefore, further rounds of self-fertilization will be needed to try and obtain homozygous 4D.

**Figure 5:**
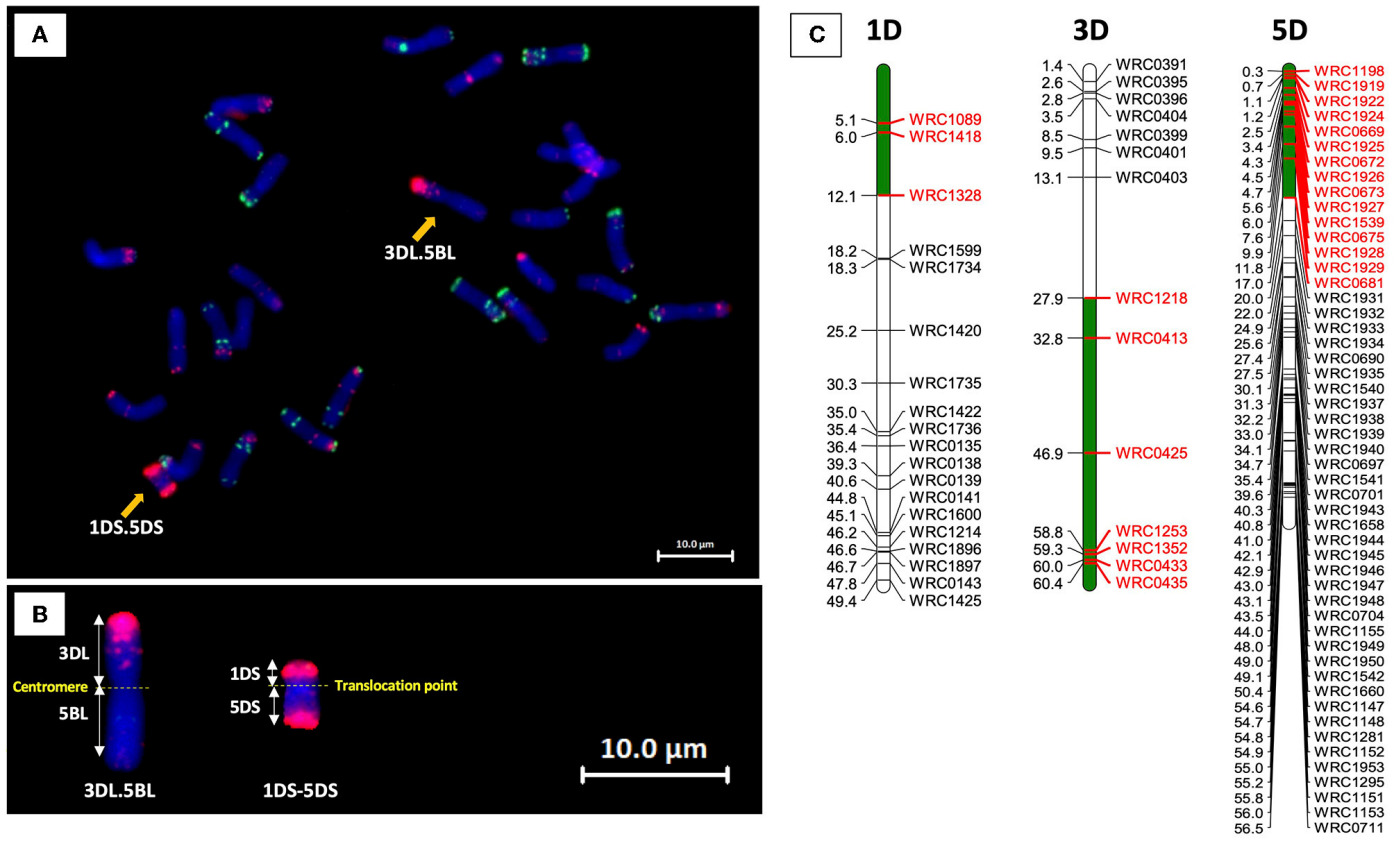
**(A)** Mc-FISH of a metaphase spread of BC_1_F_2_-250-3 showing the presence of a centromeric 3DL.5BL translocation and 1DS−5DS chromosome rearrangement (yellow arrows). **(B)** Enlarged images of the 3DL.5BL and 1DS.5DS translocated chromosomes. **(C)** KASP genotyping showing the D-genome segments in green with associated KASP markers shown in red.

The 3DL.BL translocation present in BC_1_F_1_-250 was detected in three BC_1_F_2_ progeny *via* KASP genotyping, with one of the BC_1_F_2_ plants (BC_1_F_2_-250-3) also containing the 1DS.5DS translocation. The mc-FISH analysis of BC_1_F_2_-250-3 showed that the 3DL chromosome arm had translocated with 5BL, and hence, the 3DL.BL chromosome was identified as 3DL.5BL (Figure 5). KASP analysis of all BC_1_F_3_ plants germinated from BC_1_F_2_-250-3 showed that the translocated 3DL.5BL chromosome had been lost. Progeny from the other two BC_1_F_2_ plants containing the translocated chromosome will therefore need to be germinated if this chromosome is to be obtained in a homozygous state.

### Screening for 5D in Later Generations

**Figure 6:**
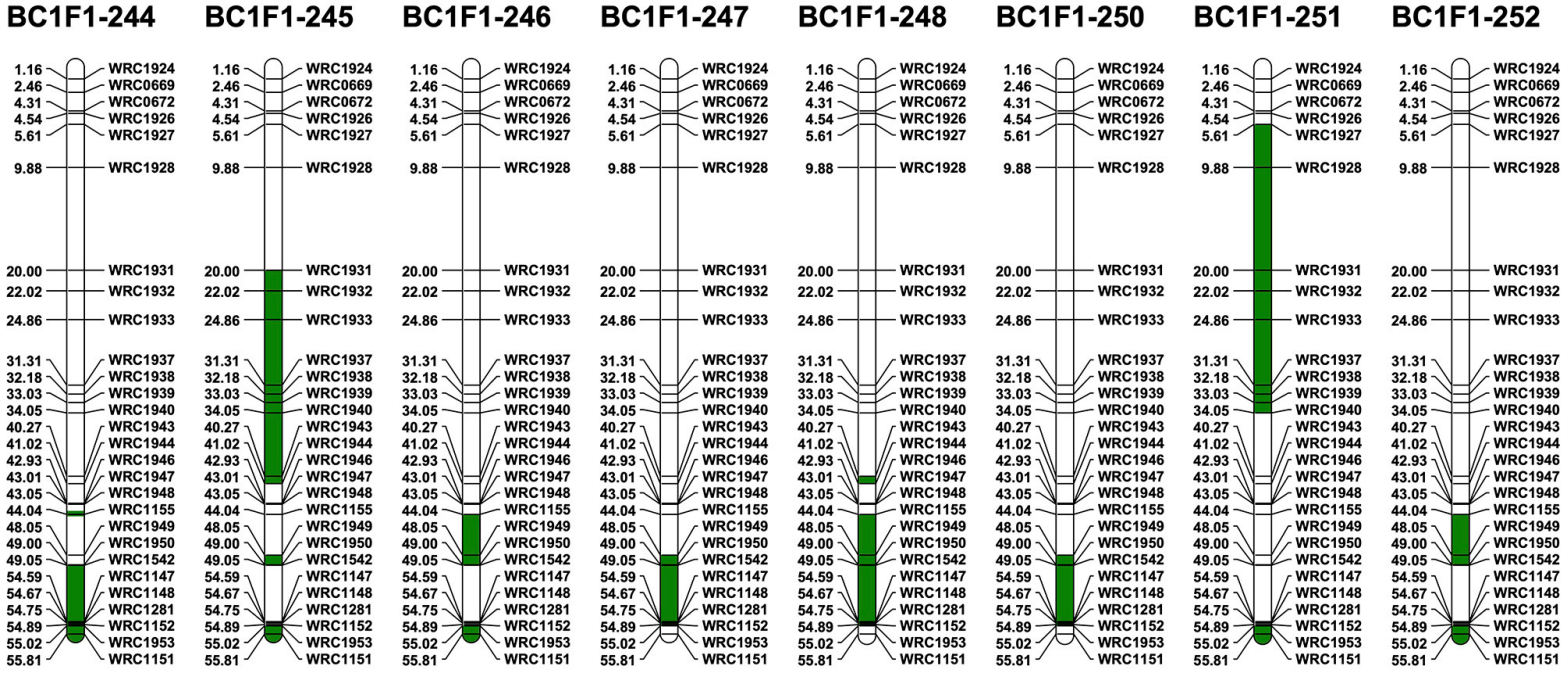
Physical position of *Ae. tauschii* segment (green) within the retained 5D chromosome in the BC_1_F_1_ lines using the 5D-chromosome-specific polymorphic KASP markers between CS and *Ae. tauschii*. Marker positions are displayed as 1 × 10^−7^ Mbp.

The 28 5D KASP markers polymorphic between Chinese Spring and *Ae. tauschii* were used to identify potential recombination between the 5D chromosomes from the two species (Figure 4). Thirty-three 5B-specific KASP markers, well-spread along the length of 5B, were also selected for use in the genotyping analysis as recombination between monosomic 5D and monosomic 5B was expected in the gametes of the F_1_T plants. The 33 markers were used (i) to help characterize breakage points between 5B and 5D and (ii) to facilitate the identification of homozygous introgressions in later generations. All eight BC_1_F_1_s were found to contain at least one 5D introgression from *Ae. tauschii* (with a maximum of three in BC_1_F_1_-245) (Figure 6). At least one recombination event between Chinese Spring 5D and *Ae. tauschii* 5D has occurred in each BC_1_F_1_, and recombination was more prevalent in 5DL with only the short arm of BC_1_F_1_-251 containing *Ae. tauschii* 5D (Figure 6). Two different large introgressions from *Ae. tauschii* were identified in BC_1_F_1_-245 and 251, while BC_1_F_1_-246 and 252 and BC_1_F_1_-247 and 250 contained the same introgressions, although in both cases the overall number of D chromosomes retained varied. Five out of the eight BC_1_F_1_s (62.5%) showed an introgression from *Ae. tauschii* at the very distal telomeric region of 5DL, marked by the amplification of marker WRC1151. Amplification of KASP marker WRC1950 showed the presence of *Ae. tauschii* in all but one of the BC_1_F_1_s, indicating that this region is within a potential hot spot for recombination.

KASP analysis of the BC_1_F_2_ progeny showed that the progeny from BC_1_F_1_-245 and BC_1_F_1_-246 had lost all D chromosomes/segments including 5D. Genotyping also showed first that the 5D(5B) monosomic substitution had been retained in progeny from the other six BC_1_F_1_ plants and second that breakage of the 5D chromosome had occurred in progeny from BC_1_F_1_-244 (one line carried a 5DS segment), BC_1_F_1_-247 (four lines carried a 5DL segment), BC_1_F_1_-251 (three lines carried the same small telomeric 5DS segment and one line carried a 5DL segment), and BC_1_F_1_-252 (one line carried a 5DL segment).

KASP and mc-FISH analyses of the four BC_1_F_2_ lines from BC_1_F_1_-247 carrying a 5DL segment showed that recombination had occurred in different places between 5DS and 5BS, and thus, these lines all contained an introgression from *Ae. tauschii*, present as a single copy in a total of 28 chromosomes. In one of the plants, recombination had occurred close to the telomere of the short arm and this plant was therefore characterized as T5BS-5DS.5DL. The remaining three lines were all found to contain a centromeric translocation between 5DL and 5BS and thus were characterized as 5BS.5DL.

**Figure 7:**
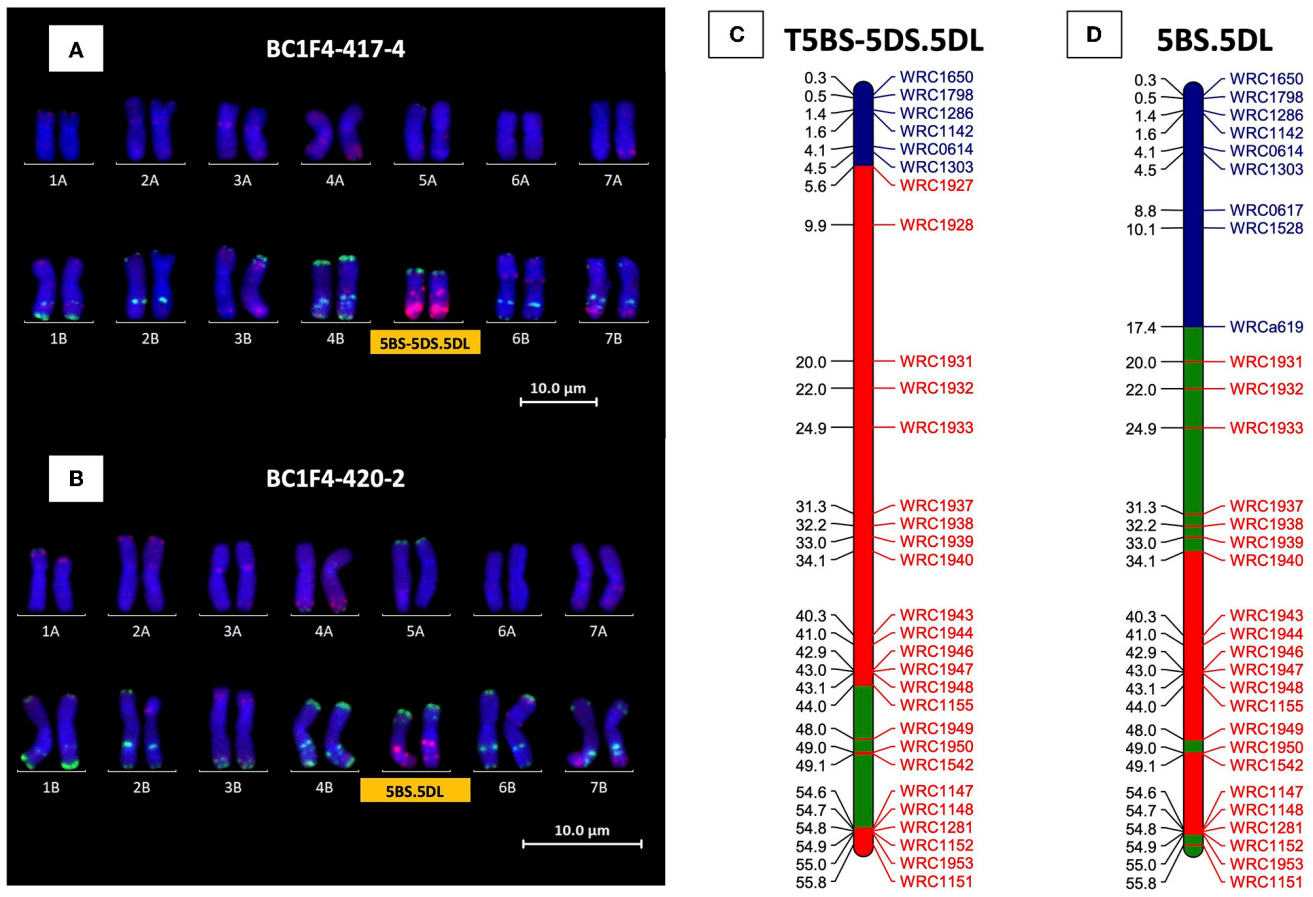
Mc-FISH-based karyotype using the Oligo-pAs.1 (red) and Oligo-pSc119.2 (green) probes counterstained with DAPI (blue) of the D-genome homozygous introgression lines **(A)** BC_1_F_4_-417-4 and **(B)** BC_1_F_4_-420-2 shown in the presence of T5BS-5DS.5DL and 5BS.5DL translocation, respectively, confirmed by the amplification of the specific KASP markers of 5BS (markers name in blue) and 5DL wheat/*Ae. tauschii* polymorphic (markers name in red) for **(C)** T5BS-5DS.5DL and **(D)** 5BS.5DL, respectively. The retained segments from 5B and 5D of wheat and 5D of *Ae. tauschii* are highlighted in blue, red, and green, respectively.

Twelve BC_1_F_3_ progeny from the line identified as containing the 5BS-5DS.5DL introgression (originally from BC_1_F_1_-247) were characterized with both 5D and 5B KASP markers in order to find a line with this introgression homozygous. Besides the amplification of the 5D KASP markers, one line, BC_1_F_3_-347-12, was found to have amplification of KASP markers for the telomeric region of 5BS only and mc-FISH confirmed the homozygous nature of the introgression. Further self-fertilization of this line generated four BC_1_F_4_ 5BS-5DS.5DL homozygous introgression lines confirmed by KASP and mc-FISH (e.g., BC_1_F_4_-417-2, Figures 7A,C).

The 5DL segments in one progeny from BC_1_F_1_-251 and one line from BC_1_F_1_-252 were characterized as centromeric translocations between 5DL and 5BS and therefore characterized as 5BS.5DL. The BC_1_F_2_ plant from BC_1_F_1_-244 containing the 5DS segment was not progressed as previous analysis had shown that BC_1_F_1_-244 did not contain any *Ae. tauschii* in the short arm of 5D (Figure 5). Genotyping analysis showed that this was also the case for the three plants produced from BC_1_F_1_-251 carrying the small telomeric 5DS segment, as the segment was outside the region of the *Ae. tauschii* introgression in this arm.

All BC_1_F_2_ lines carrying the *Ae. tauschii* introgressions in 5DL were self-fertilized in order to try and obtain homozygous introgressions. Selected lines carrying monosomic 5D(5B) were also self-fertilized to see whether breakage of chromosome 5D could be found in the next generation. The 5D(5B) substitution was found to have become homozygous in three BC_1_F_3_ progeny from BC_1_F_1_-251 where KASP markers showed the complete loss of chromosome 5B in these plants. Characterization with mc-FISH confirmed the plants were homozygous for the 5D(5B) substitution. The 5D(5B) substitution remained heterozygous in all other BC_1_F_3_ plants screened, with the exception of one BC_1_F_3_ plant from BC_1_F_1_-244, which was characterized as containing another 5BS.5DL centromeric translocation. Four different 5BS.5DL centromeric translocations were therefore identified in total having been produced from BC_1_F_1_-244, BC_1_F_1−_247, BC_1_F_1−_251, and BC_1_F_1−_252 and with each of these carrying a different *Ae. tauschii* introgression. The 5BS.5DL translocation from BC_1_F_1_-251 was found to be homozygous in only one plant of the BC_1_F_3_ generation and confirmed with KASP by a lack of amplification of chromosome arm 5BL KASP markers. A further round of self-fertilization was therefore carried out with genotyping, confirming four BC_1_F_4_ plants originating from BC_1_F_1_-251 to be homozygous for 5BS.5DL (e.g., BC_1_F_4_-420-2; Figures 7B,D). Only plants heterozygous for 5BS.5DL were found from BC_1_F_1_-244 and 252, and therefore, further self-fertilization will be required to acquire these translocations in a homozygous state.

## Discussion

A low level of crossability between *Ae. tauschii* and the tetraploid substitution line LND 5D(5B) was seen in this study with only four F_1_ hybrid seeds produced out of the 20 crosses made. Zhang et al. (2008) also reported a low crossability level of only 0.56% when crossing the LND 5D(5B) substitution line to *Ae. tauschii*, in an experiment testing the crossability level between *Ae. tauschii* and all of the Langdon D-genome disomic substitution lines. The results showed that the normal Langdon cv. carries dominant alleles on chromosomes 7A and 4B which inhibit crossability with *Ae. tauschii* (Zhang et al., 2008). The F_1_ hybrid plant showed a male-sterile phenotype. This was probably caused by the unequal chromosome number in the F_1_ hybrid which would have resulted in chromosome pairing problems at meiosis. Xu and Joppa (2000) also previously reported very low fertility to complete sterility in the F_1_ hybrid of LND 5D(5B) when crossed to either Rye or *Ae. tauschii*.

The absence of the *Ph1* locus in the F_1_ gamete promoted the occurrence of a single A-d translocation which was identified using mc-GISH in the F_1_T plant. In addition, a new centromeric translocation involving chromosomes 3D and 5B chromosome and characterized as 3DL.5BL was identified in one of the BC_1_F_1_ lines. Centromeric translocations are produced by the centromeric breakage–fusion of univalents in double monosomic plants (Sears, 1952; reviewed in Zhang et al., 2015). Hence, the univalent state of chromosomes 5B and 3D in the F_1_T plant promoted the production of the translocation. The number of univalent D chromosomes present in the F_1_T had decreased considerably in the BC_1_F_1_ generation with a maximum of three D chromosomes. At meiosis, unpaired univalent chromosomes tend to either randomly segregate or be lost. According to KASP analysis, all BC_1_F_1_ lines contained at least one 5D chromosome. FISH analysis showed that a pair of 5D chromosomes were present in the F_1_T plant. This was not expected as the two 5D chromosomes in the F_1_ plant (one inherited from LND 5D(5B) and one from *Ae. tauschii)* should have segregated into different gametes. Chromosome counting revealed that all BC_1_F_1_ lines contained only 13 B chromosomes, indicating the presence of a univalent 5B chromosome. Hence, the use of the LND 5D(5B) disomic substitution line to generate the F_1_ promoted the retention of univalent 5D and 5B chromosomes in the advanced backcross generations to durum wheat.

A higher genetic diversity is always observed in the D-genome donor *Ae. tauschii* compared to the D-genome of wheat (Reif et al., 2005), with only 25% of the genetic diversity of *Ae. tauschii* being present in hexaploid wheat (Gaurav et al., 2022). Hence, the extensive use of synthetic hexaploid wheat by breeders through the interspecific hybridization between tetraploid durum wheat and *Ae. tauschii*, to recover the genetic diversity present in the wild species. A set of seven *T. aestivum* cv. Chinese Spring/*Ae. tauschii* D chromosome substitution lines were also previously used to introgress *Ae. tauschii* into hexaploid wheat (Pestsova et al., 2001, 2006). Recombination is expected between the homologous D chromosomes of Chinese Spring and *Ae. tauschii* when the substitution lines are backcrossed to Chinese Spring, making it easier to introgress *Ae. tauschii* into bread wheat without the need of using a *Ph1* mutant line. However, it is more difficult and challenging to introgress *Ae. tauschii* into durum wheat as it lacks the D-genome. Care is also required with the introgression of segments from *Ae. tauschii* as certain genes in the D-genome can change the durum wheat grain texture and hence its end-use properties. For example, the two *puroindoline* genes, namely, *puroindoline a* (*Pina-D1*) and *puroindoline b* (*Pinb-D1*), on the distal region of chromosome arm 5DS (the *Hardness* locus) are responsible for the soft kernel trait in wheat (Bhave and Morris, 2008).

The tetraploid Langdon disomic D-genome substitution lines carry D chromosomes of Chinese Spring, and these lines can therefore be used as a bridge to introgress segments from *Ae. tauschii* into durum. In this study, the LND 5D(5B) substitution line was chosen due to the absence of the *Ph1* locus, facilitating recombination between *Ae. tauschii* and the A and B genomes of durum wheat. However, in order to track chromosome 5D from *Ae. tauschii*, it was necessary to have markers polymorphic between the 5D chromosomes of Chinese Spring and *Ae. tauschii*. For example, to track *Ae. tauschii* segments introgressed into bread wheat, Pestsova et al. (2001) used 65 microsatellite markers polymorphic between the D-genome of Chinese Spring and *Ae. tauschii* with an average of 9.3 markers per linkage group. The markers designed and used in this study will also be useful in future to transfer the *Ae. tauschii* 5D segments into a hexaploid wheat background. The publication of the genomes of wheat (International Wheat Genome Sequencing Consortium et al., 2018) and *Ae. tauschii* (Wang et al., 2021) means it is now easier to design polymorphic genome-specific markers between the D-genomes of hexaploid wheat and *Ae. tauschii* for each linkage group. Such markers will further facilitate the introgression of specific regions from *Ae. tauschii* into bread wheat and also durum wheat when the LND D-genome substitution lines are used as a bridge in the crosses.

One to three *Ae. tauschii* segments were detected on the 5D chromosome in each BC_1_F_1_ line, indicating both the number and position of crossover between the two 5D chromosomes during chromosome pairing. The introgressed *Ae. tauschii* segments were of different sizes, with segments overlapping in different lines The highest rate of recombination was observed in the long arm with the highest frequency toward the distal end as expected. Only one of the BC_1_F_1_ lines had a large segment covering the centromeric region with a recombination event in the distal region of the short arm. Several studies have shown that recombination rate increases from the centromeric region toward the telomeric region in wheat (Lukaszewski and Curtis, 1993; Erayman et al., 2004; Sidhu and Gill, 2005; See et al., 2006). In a study on the distribution of crossovers on chromosome 3B, a very similar distribution of recombination was reported by Saintenac et al. (2009) with 90% of crossovers in the distal sub-telomeric region of the long arm, whereas a low to very low frequency was observed in the distal region of the short arm and the region surrounding the centromere, respectively. The telomeric region of the 5D-chromosome long arm appeared to be a recombination hot spot. For instance, in the BC_1_F_1_ lines, a total of nine recombination events were observed in the region between markers WRC1155 and WRC1542 located at 5D:440374100 Mbp and 5D:490510565 Mbp, respectively.

The univalent state of chromosomes 5B and 5D in the BC_1_F_1_ plants promoted the occurrence of new genomic translocations involving these two chromosomes in the self-fertilized progeny at each generation. Therefore, *Ae. tauschii* introgressions were present in the homozygous 5BS-5DS.5DL and 5BS.5DL translocations. These homozygous lines were missing the long arm of chromosome 5B, and these lines will therefore act in the same way as *ph1* mutant while retaining the gene previously shown to promote pairing in the distal third of the 5B short arm (Feldman and Mello-Sampayo, 1967; Riley and Chapman, 1967; Kota and Dvorák, 1986). In addition, the long arm of chromosome 5D also carries a pairing promoter gene (Sears, 1976), and thus, the homozygous 5BS.5DL line can potentially promote more homologous pairing in wheat when compared with the disomic 5D(5B) substitution line. The frequency of genomic translocations obtained using this line could be assessed in the progeny of a crossing program to different wild relative species.

Apart from the *Ae. tauschii* tetraploid introgression lines, 5BS-5DS.5DL and 5BS.5DL, monosomic and disomic 5D substitution lines carrying different segments from chromosome 5D of *Ae. tauschii* were identified. With the use of the 5D polymorphic KASP markers, these lines can be used to introgress the *Ae. tauschii* segments present on the 5D substituted chromosome(s) into bread wheat through crossing and back crossing to hexaploid wheat.

A high retention rate of chromosomes 1D and 4D was seen in the BC_1_F_1_ generation either as monosomic additions or chromosome arm additions. However, a very low transmission rate of these univalent chromosomes was observed in the subsequent generation of self-fertilization. In general, monosomic additions in wheat have been shown to have a low transmission rate after plant self-fertilization (Makino, 1981; Dhaliwal et al., 1990). In addition, monosomic chromosomes frequently undergo centromeric breakage producing telocentrics that are mainly lost through meiosis. For example, centromere breakage and fusion were shown to occur in the F_1_s of wheat–rye hybrids (Lukaszewski and Gustafson, 1983; Friebe and Larter, 1988). A rearranged 1DS.5DS chromosome was identified in one of the BC_1_F_2_ lines in this study. However, this rearrangement would have occurred here in the gametes of the BC_1_F_1_ generation as 5D was present as a chromosome pair in both the F_1_ hybrid and the F_1_T plant. Both KASP markers and mc-GISH showed the presence of univalent 1D and 5D chromosomes in the BC_1_F_1_ making them prone to mis-division followed by fusion and leading to the rearrangement. Despite its low retention rate after plant self-fertilization, the 1DS−5DS chromosome was retained in one of the BC_1_F_4_ lines as a monosomic addition although no line was found where it was homozygous.

The overall results of this study showed that the use of the LND 5D(5B) substitution line to generate durum wheat/*Ae. tauschii* introgression lines did not promote the D-genome introgression across all linkage groups as expected. Chromosomes 6D and 7D were not found in any of the BC_1_F_1_ lines, chromosome 2D was lost by the BC_1_F_2_ generation, and although chromosome 3DL was found in a BC_1_F_1_ line as a translocation with 5BL (3DL.5BL) and was transmitted to the BC_1_F_2_ generation, it was not transmitted to the BC_1_F_3_ generation. A higher retention was seen with chromosomes 1D and 4D in the early generations, but transmission through later generations was very low with only a 1DS.5DS monosomic addition, a 1DL monotelosomic addition, and a 4D monosomic addition retained in the BC_1_F_4_ generation. Translocations between the 5D and 5B chromosomes occurred in the self-fertilized progeny of the 5D(5B) monosomic lines, leading to the introgression of *Ae. tauschii* 5D chromosome segments recombined with the 5D of wheat into a tetraploid wheat background. Five lines were identified: a 5BS-5DS.5DL translocated chromosome and four 5BS.5DL centromeric translocations. The 5BS-5DS.5DL chromosome and two of the 5BS.5DL chromosomes were found to be homozygous in the BC_1_F_4_ generation, but the other two 5BS.5DL chromosomes remained heterozygous.

The LND 5D(5B) substitution line was used in this work as the absence of *Ph1* was expected to promote D-genome introgression across all linkage groups. However, only a limited level of success was achieved for linkage group 5D. It is possible that the use of the LND substitution lines for other linkage groups may result in better levels of introgression of the D-genome from *Ae. tauschii* for those linkage groups, for example, more introgression of *Ae. tauschii* linkage group 1 with the use of LND 1D(1B).

The 5BS-5DS.5DL and 5BS.5DL homozygous lines are currently being multiplied and will be deposited at the Germplasm Resource Unit at the John Innes Centre. The KASP markers designed in this program will be useful in future programs targeting the transfer of genetic variation from chromosome 5 of *Ae. tauschii* into hexaploid wheat.

## Data Availability Statement

The original contributions presented in the study are included in the article/Supplementary Material, further inquiries can be directed to the corresponding author/s.

## Author Contributions

MO carried out the crossing program. MO, SG, and JW contributed to the KASP genotyping. MO, JW, and C-yY contributed to the GISH analysis. MO, IK, JK, and SG contributed to the conceptualization and manuscript writing. IK and JK contributed to the funding acquisition. All authors have read and agreed to the published version of the manuscript.

## Funding

This research was supported by the Monsanto Beachell-Borlaug International Scholarship program (2015) and by the Biotechnology and Biological Sciences Research Council [Grant Nos. BB/J004596/1 and BB/P016855/1] as part of the Wheat Improvement Strategic Programme (WISP) and Designing Future Wheat (DFW) Programme, respectively.

## Conflict of Interest

The authors declare that the research was conducted in the absence of any commercial or financial relationships that could be construed as a potential conflict of interest.

## Publisher's Note

All claims expressed in this article are solely those of the authors and do not necessarily represent those of their affiliated organizations, or those of the publisher, the editors and the reviewers. Any product that may be evaluated in this article, or claim that may be made by its manufacturer, is not guaranteed or endorsed by the publisher.
