# Supplementary material for: Assessing the Potential of Using the Langdon 5D(5B) Substitution Line for the Introgression of Aegilops tauschii Into Durum Wheat
Source: Front Plant Sci. 2022 Jul 7;13:927728. doi: 10.3389/fpls.2022.927728 (PMC9302120; doi:10.3389/fpls.2022.927728)
Supplement: Supplementary file 1 [file Data_Sheet_1.PDF]

BC1F1-244

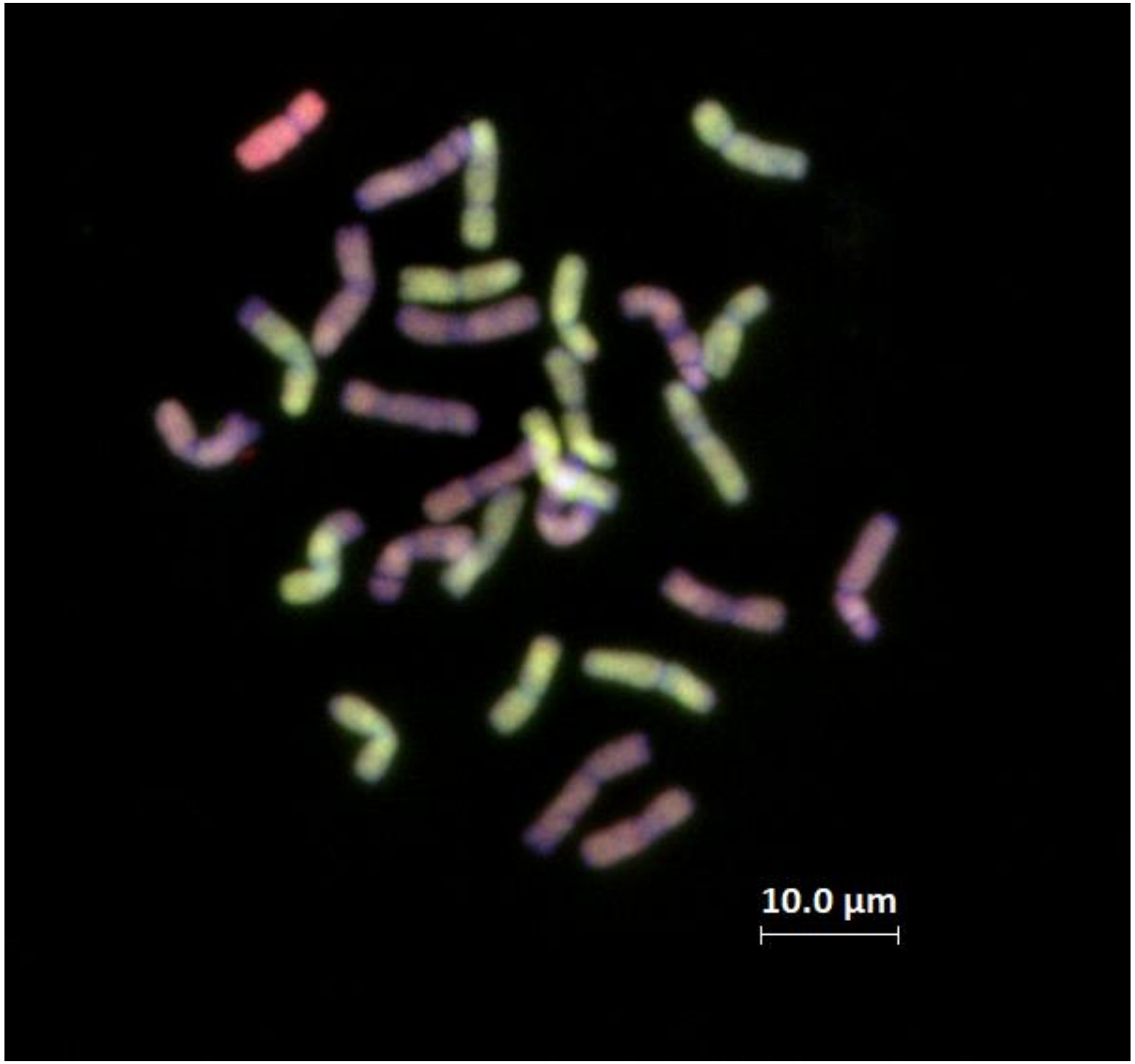

Chromosomes colour code:

A-genome: Green

B-genome: Blue/purple

D-genome: Red

BC1F1-245

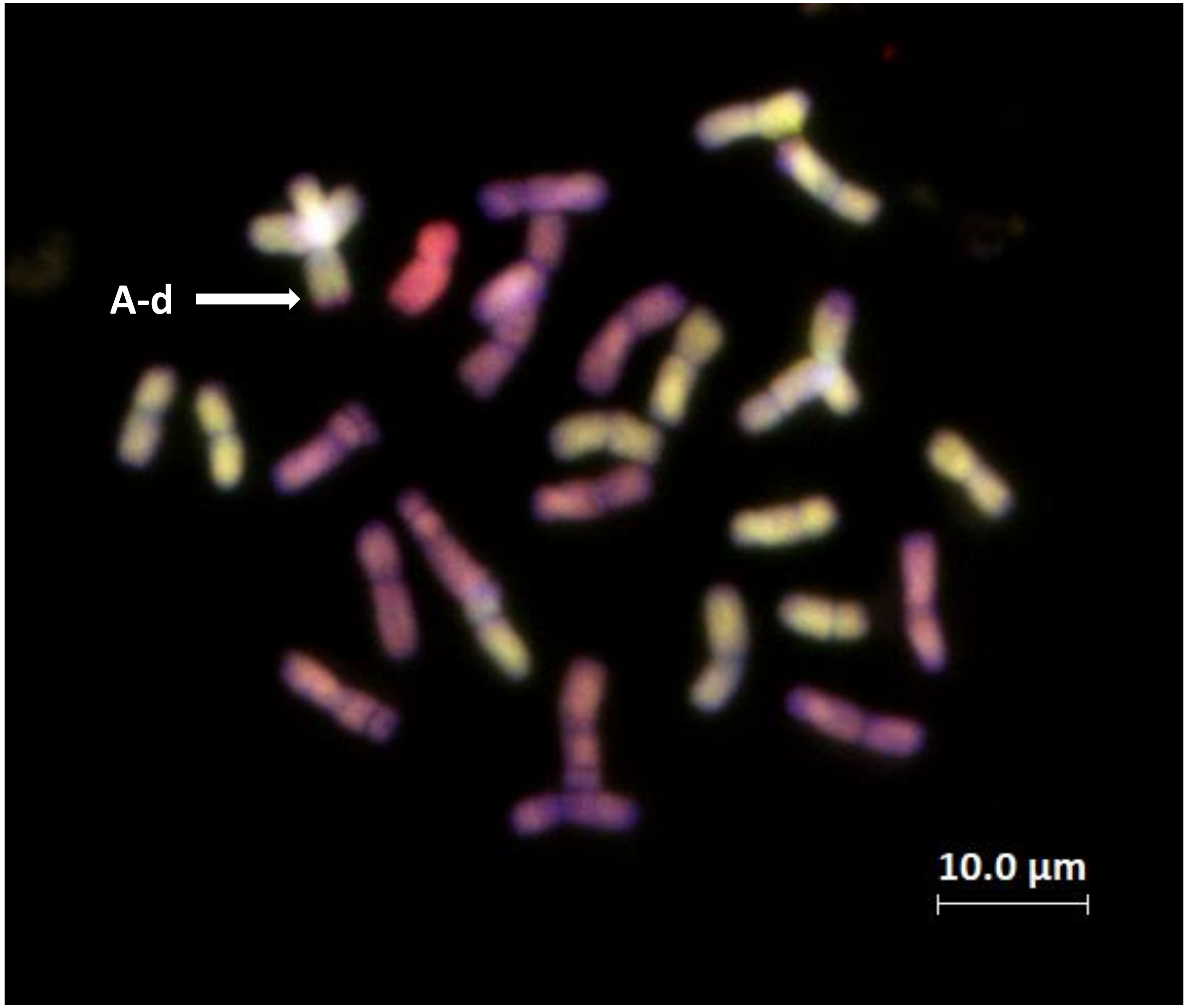

<u>Chromosomes colour code:</u>

A-genome: Green

B-genome: Blue/purple

D-genome: Red

BC1F1-246

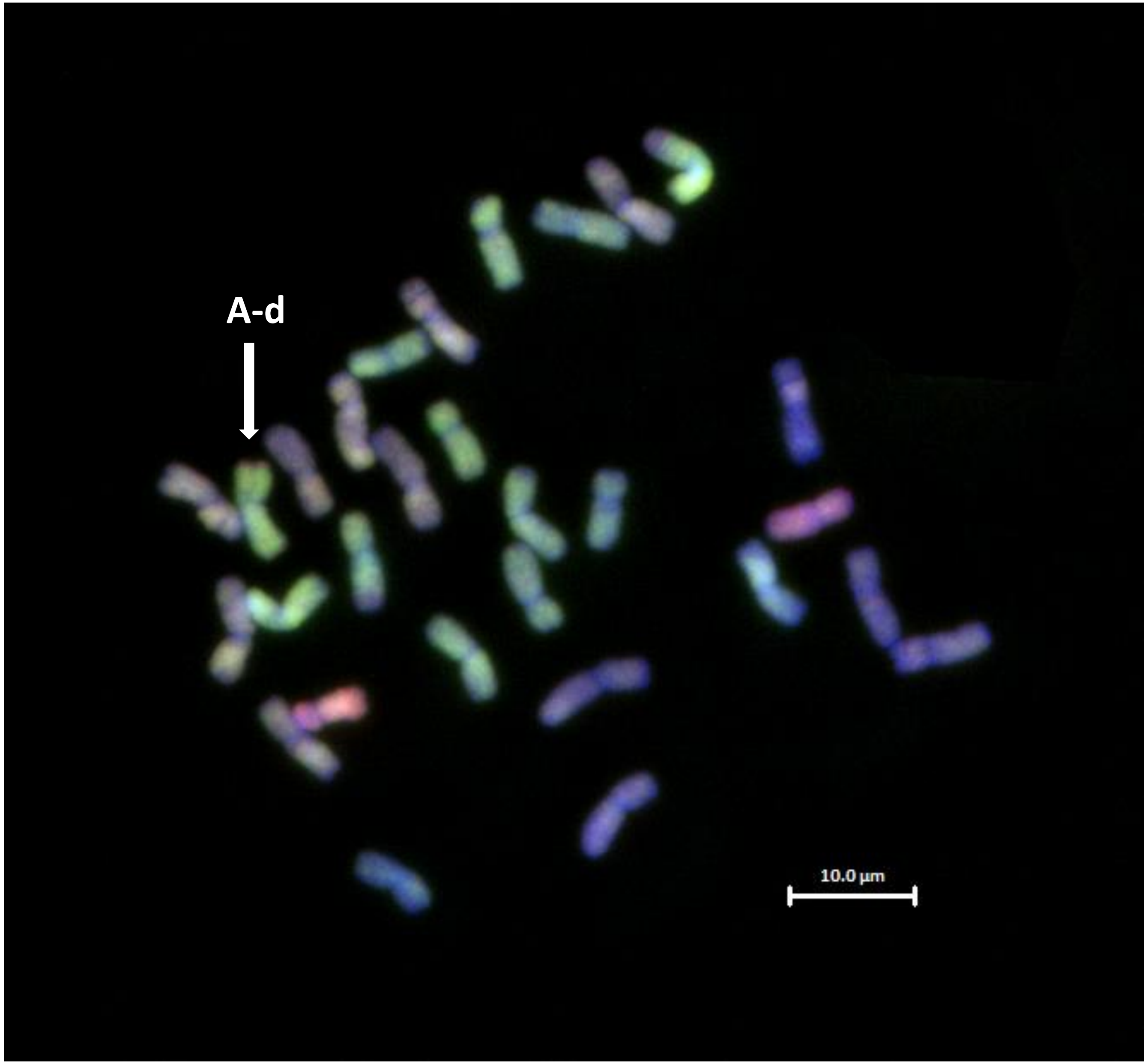

Chromosomes colour code:

A-genome: Green

B-genome: Blue/purple

D-genome: Red

BC1F1-247

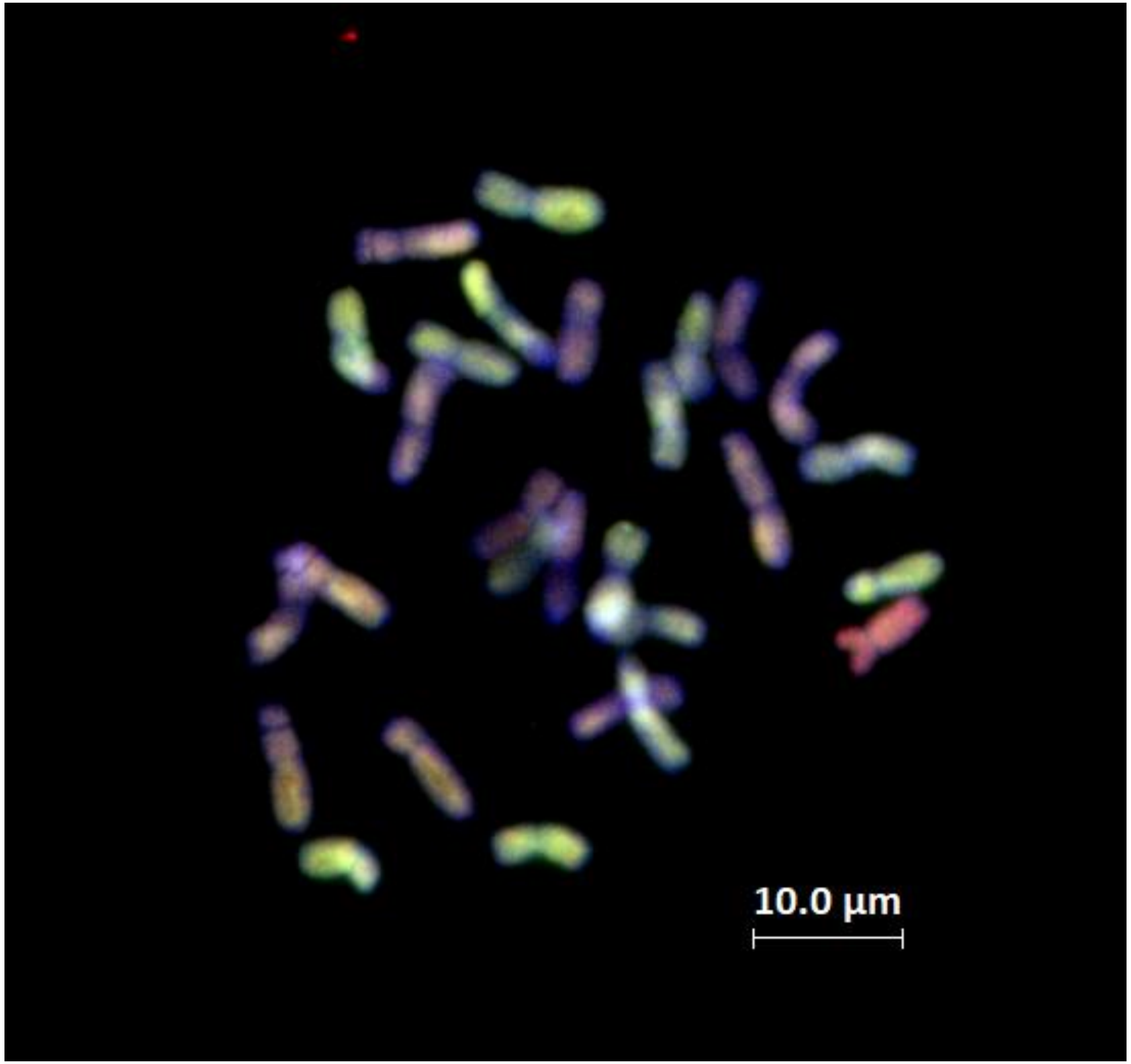

Chromosomes colour code:

A-genome: Green

B-genome: Blue/purple

D-genome: Red

BC1F1-248

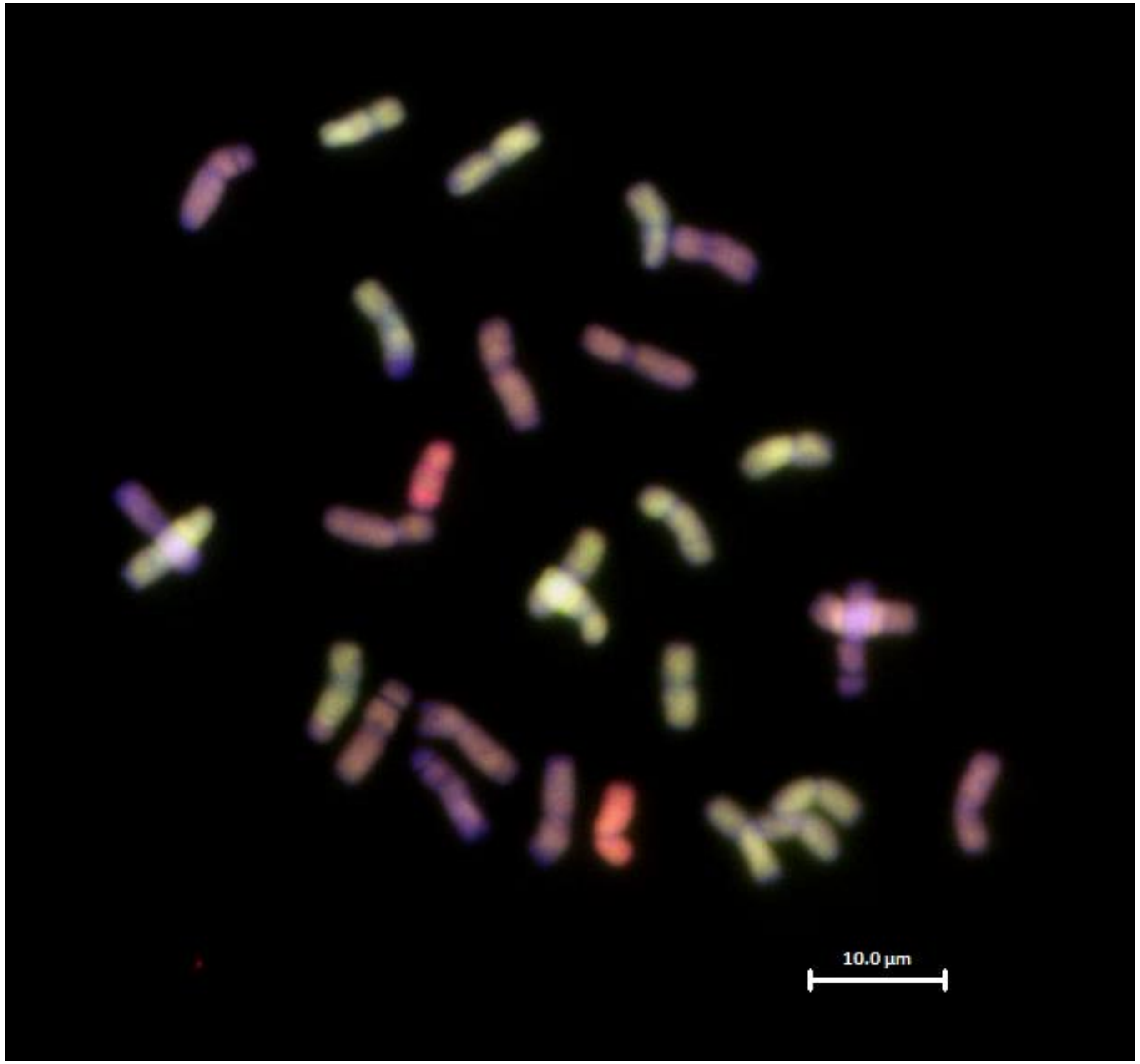

Chromosomes colour code:

A-genome: Green

B-genome: Blue/purple

D-genome: Red

BC1F1-250

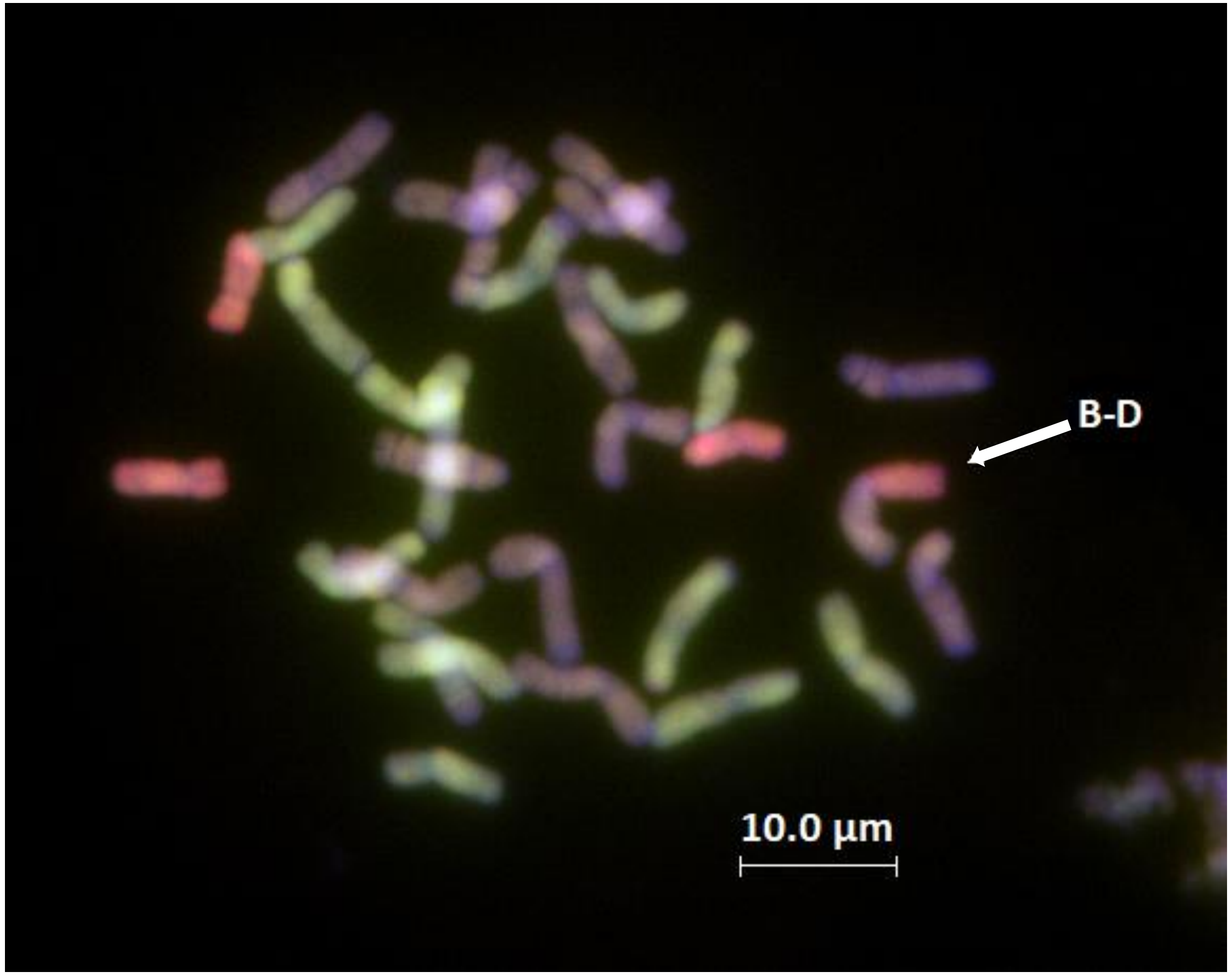

Chromosomes colour code:

A-genome: Green

B-genome: Blue/purple

D-genome: Red

BC1F1-251

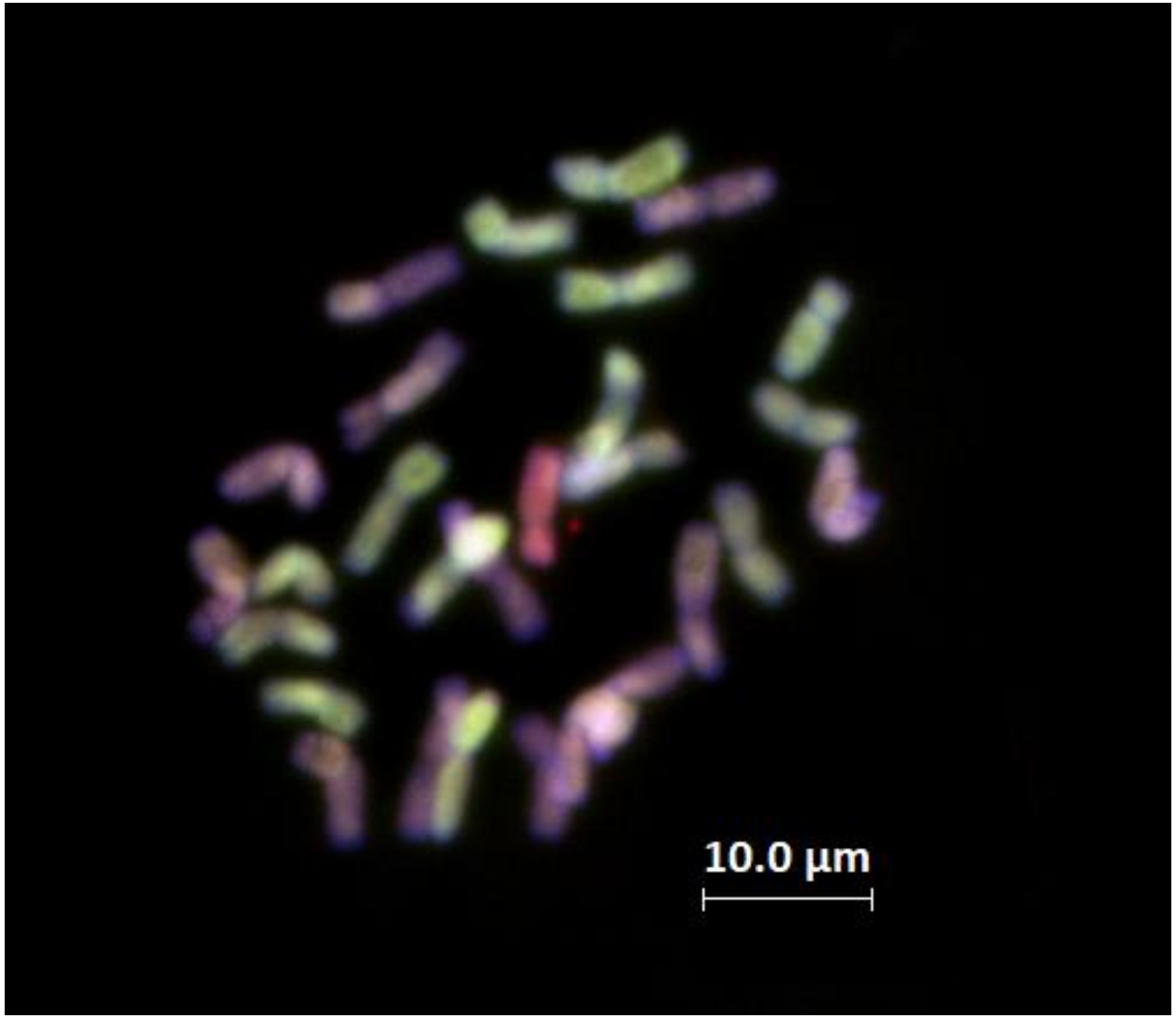

Chromosomes colour code:

A-genome: Green

B-genome: Blue/purple

D-genome: Red

BC1F1-252

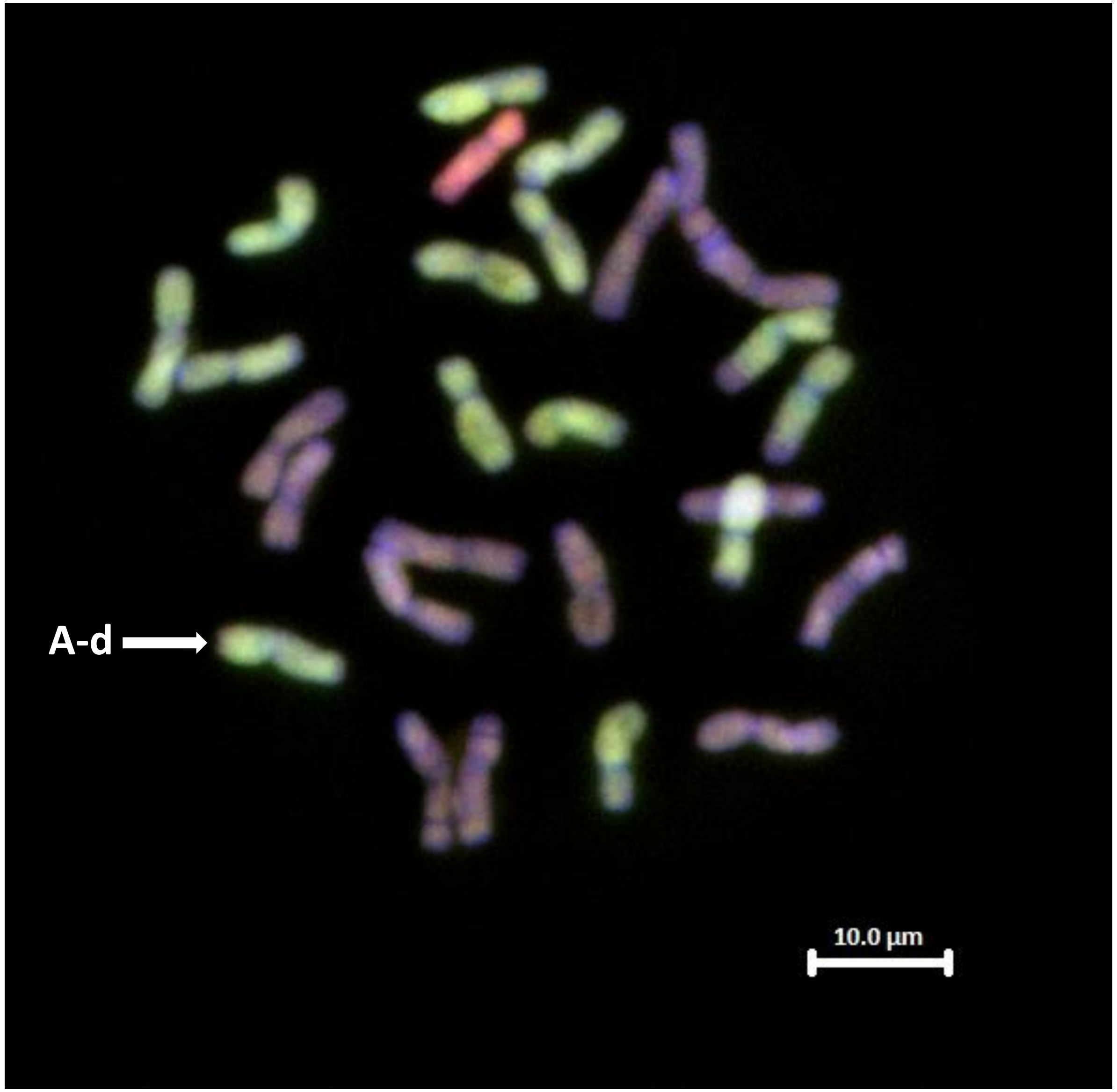

Chromosomes colour code:

A-genome: Green

B-genome: Blue/purple

D-genome: Red
